# Highly Variable Sialylation Status of Donor-Specific Antibodies Does Not Impact Humoral Rejection Outcomes

**DOI:** 10.3389/fimmu.2019.00513

**Published:** 2019-03-20

**Authors:** Thomas Barba, Jean Harb, Stéphanie Ducreux, Alice Koenig, Virginie Mathias, Maud Rabeyrin, Eric Pouliquen, Antoine Sicard, Dimitri Chartoire, Emilie Dugast, Thierry Defrance, Emmanuel Morelon, Sophie Brouard, Valérie Dubois, Olivier Thaunat

**Affiliations:** ^1^French National Institute of Health and Medical Research (INSERM) Unit 1111, Lyon, France; ^2^French National Institute of Health and Medical Research (INSERM) UMR1064, Nantes, France; ^3^Laboratory of Biochemistry, Nantes University Hospital, Nantes, France; ^4^French National Blood Service (EFS), HLA Laboratory, Lyon, France; ^5^Department of Transplantation, Nephrology and Clinical Immunology, Hospices Civils de Lyon, Edouard Herriot Hospital, Lyon, France; ^6^Lyon-Est Medical Faculty, Claude Bernard University (Lyon 1), Lyon, France; ^7^Department of Pathology, Hospices Civils de Lyon, Bron, France

**Keywords:** antibody-mediated rejection, DSA, sialylation, glycosylation, solid organ transplantation

## Abstract

Clinical outcome in antibody-mediated rejection (AMR) shows high inter-individual heterogeneity. Sialylation status of the Fc fragment of IgGs is variable, which could modulate their ability to bind to C1q and/or Fc receptors. In this translational study, we evaluated whether DSA sialylation influence AMR outcomes. Among 938 kidney transplant recipients for whom a graft biopsy was performed between 2004 and 2012 at Lyon University Hospitals, 69 fulfilled the diagnosis criteria for AMR and were enrolled. Sera banked at the time of the biopsy were screened for the presence of DSA by Luminex. The sialylation status of total IgG and DSA was quantified using *Sambucus nigra* agglutinin-based chromatography. All patients had similar levels of sialylation of serum IgGs (~2%). In contrast, the proportion of sialylated DSA were highly variable (median = 9%; range = 0–100%), allowing to distribute the patients in two groups: high DSA sialylation (*n* = 44; 64%) and low DSA sialylation (*n* = 25; 36%). The two groups differed neither on the intensity of rejection lesions (C4d, ptc, and g; *p* > 0.05) nor on graft survival rates (Log rank test, *p* = 0.99). *in vitro m*odels confirmed the lack of impact of Fc sialylation on the ability of a monoclonal antibody to trigger classical complement cascade and activate NK cells. We conclude that DSA sialylation status is highly variable but has not impact on DSA pathogenicity and AMR outcome.

## Introduction

Progresses achieved over the last decades in the field of renal transplantation have not significantly improved graft survival (1). Epidemiological studies have identified antibody-mediated rejection (AMR) as the main cause of renal allograft failure (2–4).

The pathophysiological sequence of AMR starts within recipient's secondary lymphoid organs (5), in which a T cell-dependent humoral response against mismatched HLA molecules leads to the generation of switched, high affinity, donor-specific antibodies (DSA) (6). Because of their size, IgG are largely retained in the circulation (7). Graft vasculature therefore represents the biological interface between donor alloantigens and host DSA. The binding of circulating DSA to directly accessible targets expressed by graft endothelial cells sometimes activate the classical complement pathway (8, 9) but this mechanism is not mandatory for the development of histological lesions (10, 11). Engagement of the surface Fc receptors of innate immune effectors (including neutrophils, monocytes, and NK cells) by DSA bound to graft microvasculature is indeed sufficient to trigger the release of lytic enzymes, a process named antibody dependent cell cytotoxicity (ADCC) (11, 12).

Following renal transplantation, ~25% of patients develop *de novo* DSA within 5 years (4, 13). While, at the population level, the presence of circulating DSA strongly correlates with an increased risk for graft loss (2–4), it has long been observed that some patients maintain long-term graft function after AMR (9). This suggests that DSA may be heterogeneous with regard to their pathogenicity.

The first identified parameter associated with DSA pathogenicity was the titer, as assessed by the MFI value in single antigen assays (14, 15). More recently, our group (9) and others (8) have reported that it was possible to stratify the risk of graft loss for renal transplant patients with DSA on the basis of *in vitro* tests that measure the ability of alloantibodies to bind complement proteins. Although activation of classical complement pathway largely depends on antibody titer (16), subgroup analysis showed that the predictive value of these assays was independent of DSA MFI (9), suggesting that qualitative characteristics of DSA might impact on their pathogenicity.

Ig molecule is shaped like a Y, with two identical halves, each made up of a heavy chain and a light chain. The 2 arms of the Y, each formed by the amino terminal extremity of a heavy chain and a light chain, contain the antigen-binding site (Fab). The base of the Y, composed by the carboxy terminal extremity of the constant region of the two heavy chains, is named fragment crystallizable (Fc). By binding to Fc receptors on immune effectors and to complement proteins, the Fc region confers to the Ig molecule its effector functions. It seems therefore reasonable to speculate that the characteristics of the Fc fragment influence DSA pathogenicity.

**Figure 1:**
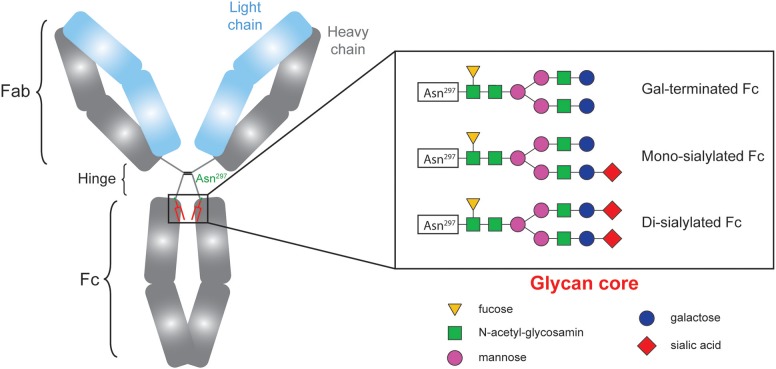
The sialylation status of immunoglobulins Fc fragment is variable.

CH2 domains of the Fc fragment of IgG contain complex oligosaccharide structures covalently attached to asparagine 297 (17–19). The bi-antennary core glycan structure, which is composed of 2 N-acetyl-glucosamines (GlcNAc) and 3 mannoses, can be further modified with fucose, bisecting GlcNAc and terminal GlcNAc, galactose, and sialic acid (Figure 1). The sialylation status of the Fc region might be important since some studies have demonstrated that increased Fc sialylation results in reducing the affinity of IgG molecules for pro-inflammatory Fcγ receptors (20, 21) and could impair their ability to trigger complement-dependent cytotoxicity (22).

Based on these data we initiated this translational study aiming at evaluating whether the sialylation status of the DSA Fc region could modulate their pathogenicity and therefore influence humoral rejection outcomes.

## Methods

### Study Population

The study was carried out in accordance with French legislation on biomedical research and the Declaration of Helsinki.

The reports of all kidney allograft biopsies performed between September 1, 2004, and September 1, 2012, at Edouard Herriot Hospital or Jules Courmont Hospital, the two university hospitals in Lyon, France, were screened (2,024 biopsies in 938 patients) by means of the pathology department's computer database (DIAMIC). All kidney transplant recipients who displayed DSAs during the same period were identified through the immunology department's computer database. Information from the two databases was compared to identify patients with microvascular inflammation (defined as g+ptc Banff scores ≥2) and concomitant DSA, thus fulfilling the diagnosis criteria of AMR according to Banff classification.

A renal pathologist and a nephrologist reviewed all biopsy specimens. Serum samples banked at the time of biopsy (N° of biocollection: AC- 2011-1375 and #AC-2016-2706), were retested using the same batch of the same Single Antigen Flow Beads assay. Sixty-nine patients were finally enrolled in the study. Last follow up was set at September 30, 2017. Mean follow-up after AMR ± standard deviation was 40.8 ± 36.2 months.

All patients received an ABO-compatible transplant with negative historical and current complement-dependent cytotoxicity crossmatches.

### Detection and Characterization of DSA

All the analyses were performed in a blinded fashion by a trained immunobiologist (VD). HLA typing of donors and recipients was performed by PCR SSO reverse (One Lambda, Canoga Park, CA). Serum samples were analyzed using Single Antigen Flow Beads assays (LSA class I and class II, Immucor, Norcross, GA). The MFI was measured on a LABscan IS 200, and all the specificities with MFI > 500 and AD-BCR > 5 were considered positive (AD-BCR is MFI adjusted to the quantity of coated antigen per bead).

Serum samples were analyzed in a blinded fashion for the presence of C3d-binding donor-specific anti-HLA antibodies with the use of single-antigen flow bead assays according to the manufacturer's protocol (C3d-binding antibody assay; Immucor).

### Purification of Total IgGs From Serum Samples

Fifty microliter of Protein G Sepharose 4 Fast Flow (GE Healthcare, Uppsala, Sweden) were washed three times with 300 μl of 20 mM phosphate buffer pH 7.0. Gel was centrifuged after each wash at 8,000 × g for 5 min. Patients sera were then diluted to 200 μl with 20 mM phosphate buffer and added to the Protein G Sepharose gel and incubated overnight at 4°C. Gel was then centrifuged at 8,000 × g for 5 min and washed three times with 300 μl of phosphate buffer. IgGs were then eluted twice by adding 200 μl of 100 mM glycine pH 2.8 for 5 min and the eluate was recovered by centrifugation at 10,000 × g for 5 min and immediately neutralized at pH 7.4 by adding 8 μl of 1M Tris-HCl pH 9.0. Both elutions were pooled and the protein content was estimated by nanodrop measurement.

### Sambucus Nigra Agglutinin-Based Chromatography

SNA Sepharose (Glycodiag, Orléans, France) was washed thrice with 50 mM Tris-HCl, 150 mM NaCl buffer pH 7.5 (TBS) and CaCl2 1 mM. A 10-fold concentrated Ca-containing TBS buffer pH7.5 (500 mM Tris-HCL, 1.5M NaCl and CaCl2 10 mM) was diluted 1:10 in the 420 μL fraction containing the total purified IgGs and the solution was added to the SNA Sepharose gel and incubated overnight at 4°C. The gel was then centrifuged at 8,000 × g for 5 min. Di-sialylated IgGs were then eluted by adding 100 mM glycine pH 2.8 for 5 min and the eluate was recovered by centrifugation at 10,000 × g for 5 min and immediately neutralized at pH 7.4 with 1M Tris-HCl pH 9.0. A second elution was performed to ensure the recovering of total di-sialylated IgGs (23) bound to the gel. Both elutions were pooled and the protein content was estimated by spectrophotometry at 280 nm using nanodrop ND-1000 (Wilmingto, DE, USA). The flow-through (i.e., un/mono-sialylated fraction) and the elution fraction (i.e., di-sialylated fraction) for each purified patient serum were then dialyzed against PBS and concentrated on an Amicon Ultra-0.5 ml (3K) centrifugal filters (Millipore, Tullagreen, IRL) as recommended by the manufacturer. Each concentrated fraction was adjusted to the same final volume.

### Quantification of Total IgG Sialylation Rate

An ELLA (Enzyme Linked Lectin Assay) was used for total IgG sialylation detection. 96-well plates (NuncMaxiSorp™) were coated overnight at 4°C with 50 μL donkey anti-human IgG antibody (1.2 mg/mL) diluted in 25 mM borate buffer pH 9 (Jackson ImmunoResearch, West Grove, USA). After washes in 300 μL PBS Tween 0.05% PW40 (Sanofi Pasteur Diagnostics, Lyon, France), 100 μL periodic acid (5 mM) per well was added for 10 min at room temperature, protected from light to oxidize the coating antibody sialic acids. The plates were then saturated with 100 μL bovine gelatin B grade (Sigma, Saint Louis, USA) 0.25% in PBS Tween 0.01%, at 37°C, for 2 h. After washes, 50 μL of serum diluted at 1/10000 in PBS Tween 0.1% are deposited in duplicate and incubated for 2 h at 37°C. Sialic acid on Fc fragment was revealed with 50 μL biotinylated SNA lectin (2 μg/mL) (Glycodiag, Orléans, France) for 1 h 30 min and then 50 μL streptavidin HRP (1/2000 dilution) (Jackson ImmunoResearch. After washes, 50 μL chromogenic substrate for the HRP, TMB (Roche, Bale, Switzerland) was added for 5 min and reaction stopped by 50 μL sulfuric acid (H_2_SO_4_)_._ Optical densities (OD) were read using a plate reader (MRX) at 450 nm.

ELISA (Enzyme Linked Immuno Sorbent Assay) was used in parallel for total IgG detection. Plates were coated, treated with periodic acid and saturated as for the ELLA test. Fifty microliter of same serum diluted at 1/300000 in PBS Tween 0.1% are deposited in duplicate and incubated for 2 h at 37°C. IgG binding was revealed by incubating the plates with 50 μL mouse anti-human kappa chain antibody (1/10000 dilution, in-house production) for 1 h 30 min and 50 μL donkey anti-mouse (H+L)-HRP (1/2000 dilution) (Jackson ImmunoResearch, West Grove, USA) for 1 h. After washes, 50 μL chromogenic substrate for the HRP, TMB (Roche, Bale, Switzerland) was added for 5 min and reaction stopped by 50 μL sulfuric acid (H_2_SO_4_). OD(SNA)/OD(IgG) ratio was divided by 30 to take into account of the dilution factor of IgG for SNA and total IgG detection and was used as an approximation of the total IgG sialylation rate.

### Quantification of DSA Sialylation Rate

Flow through and eluted fractions were analyzed for their DSA content using Single Antigen Flow Beads assays as described above using LSA class I and class II (Lifecodes, Norcross, GA).

Most patients had several DSA at various titers. The proportion of di-sialylated DSA was obtained for each specificity of each patient by dividing the mean fluorescence intensity measured for the di-sialylated fraction by the sum of MFI measured for the same bead in un/mono-sialylated and di-sialylated fractions. In median the proportion of di-sialylated DSA was 9% (range: 0–100%).

For each patient, the mean proportion of di-sialylated DSA was calculated using ponderation according to the MFI of each DSA. Patients were classified in high DSA sialylation group if the ponderated mean of di-sialylated DSA was above 9%.

### Statistical Analysis

Categorical variables were expressed as percentages and compared with the chi-squared test. Continuous variables were expressed as mean ± SD and compared using the *t*-test.

Graft survival was calculated from the date of AMR diagnosis until the beginning of hemodialysis. Survival curves were constructed with the Kaplan-Meier method and compared with the log-rank test.

The Cox proportional hazards regression model was used in both univariate and multivariate models. All significant variables in the univariate analysis with a level set at *p* < 0.1 were incorporated into multivariate models. All tests were two sided, and *p* < 0.05 were considered to represent statistically significant differences. Analyses were carried out using R software version 3.5.1 (R Foundation for Statistical Computing, Vienna, Austria, 2018, https://www.R-project.org/).

### *In vitro* Study

#### Chemoenzymatic Glycosylation of Rituximab

All substrates and enzymes are from Sigma Aldrich (St Quentin Fallavier, France). Six hundred microgram of Rituximab (RTX) were incubated in 50 mM MOPS buffer pH 7.5 in presence of MnCl_2_ 5 mM, uridine mono-phosphate (UDP) galactose 0.3 mM and bovine milk galactosyl transferase 125 mU at 37°C for 65 h. The galactosylated Rituximab (Gal-RTX) was then divided in two parts and incubated or not with 3 mM cytidine di-phosphate (CDP) acid sialic and 250 mU of α-2,6 sialytransferase from *Photobacterium damsela* at 37°C for 65 h (Sial-RTX). Fractions were then dialyzed against PBS pH 7.4 and concentrated on an Amicon Ultra-0.5 ml (100 kD) centrifugal filters (Millipore, Tullagreen, IRL) as recommended by the manufacturer. The final volume of each concentrated fraction was about 200 μl.

#### Competitive Binding Assay

CD20-positive GRANTA cells derive from leukemic transformation of mantle cell lymphoma (24). 10^5^ GRANTA cells were incubated in 100 μl of FACS buffer (PBS, 1% BSA) in increasing concentrations of AF488-conjugated rituximab (a chimeric mouse-human IgG1 monoclonal antibody directed against human CD20). After 30 min at 4°C, cells were washed, and their MFI was measured on a LSR FORTESSA (BD biosciences). Plateau of fluorescence, indicating the saturation of surface targets, was obtained for 1 μg of rituximab (data not shown).

To compare the ability of the different glycosylated forms of rituximab to bind to CD20, 10^5^ GRANTA cells were first incubated with 2 μg of either Ctrl-RTX, Gal-RTX, or Sial-RTXi for 30 min at 4°C. Cells were then washed and incubated in 2 μg of AF488-conjugated rituximab. The reduction of MFI observed as compared with GRANTA cells incubated directly in AF488-conjugated rituximab was expressed as a percentage.

#### Complement-Dependent Cytotoxicity Model

The model is derived from the work by Ferreira et al. (25) Briefly, 2.10^6^ GRANTA cells/ml were placed at 4°C for 20 min in RPMI-1640 containing Penicillin/Streptomycin in presence of monoclonal antibody anti-CD55 (clone BRIC216; Millipore) and anti-CD59 (Clone MEM-43; Thermofisher) able to block human complement regulatory molecules CD55 and CD59 (both mAb at final concentration of 5 μg/mL). Cells were washed twice with PBS and 1.10^5^ cells were placed at 37°C and 5% CO_2_ in 96 well V bottom plates in presence or absence of RTX for 30 min. Human serum complement (TECOmedical) was then added to a final concentration of 10%. After 12 h at 37°C, cells were washed and resuspended in 200 μl PBS. DAPI (Sigma) stain was added 1 μl/well together with 3.10^3^ Sphero accucount (Spherotech: 10.4 μm). A thousand beads were acquired in a FACS Canto II (BD Biosciences) and the number of viable (DAPI-negative) GRANTA cells was enumerated with FlowJo® software (FlowJo, LLC).

#### Fcγ-Receptor Dependent NK Cell Activation Model

Human peripheral blood mononuclear cells (PBMC) were isolated from the blood of healthy volunteers by ficoll (density 1,077) and resuspended in RPMI-1640 containing Penicillin/Streptomycin and 10% heat inactivated pooled human serum and incubated overnight at 4°C.

2.10^5^ GRANTA cells were incubated for 30 min at 37°C in presence of the indicated amount of the one of the 3 glycoforms of RTX. 2.10^5^ PBMCs were then added and the co-culture was carried out for 4 h. Twenty microliter of GOLGI STOP (BD Bioscience) were added in each well for the last 3 h of culture. After washing in PBS, the cells were stained for 30 min at 4°C with a cocktail of fluorescent mAb: Anti-CD3 APC AF750 (Clone UCHT1; Beckman), anti-CD107a FITC (Clone eBioH4A3; eBioscience), anti-CD56 APC (Clone NCAM16.2), anti-CD19 PerCP-Cy5.5 (Clone HIB19), anti-CD14 PerCP Cy5.5 (Clone M5E2), anti-CD4 PerCP Cy5.5 (Clone SK3) all from BD and a viability Dye eFluor 506 (BD). Then the cells were washed and permeabilized with Cytofix/Cytoperm (BD) to allow for staining of MIP-1β (anti-MIP-1β V450, Clone D21-1351; BD). Flow cytometry analysis was performed with a FACS Canto II and data were analyzed with FlowJo® software.

## Results

### Characteristics of the Study Population

Of the 938 kidney transplant recipients followed in our institutions between September 1st 2004 and September 1st 2012, 69 (7.3%) fulfilled the diagnostic criteria for AMR (i.e., sum of the Banff scores for glomerulitis and peritubular capillaritis ≥2 and presence of circulating anti-HLA DSA) and were enrolled in the study. Tables 1, 2 summarize the characteristics of the study population.

**Table 1:** Patient characteristics at baseline.

**Variable**	**Patients with** **AMR (*n* = 69)**	**Patients with** **di-sialylated DSA** **(*n* = 44)**	**Patients with un/mono** **sialylated DSA** **(*n* = 25)**	*p*-Value^*^
**CHARACTERISTICS AT THE TIME OF TRANSPLANTATION**				
**Recipient**				
Gender, male, *n* (%)	42 (60.9)	24 (54.5)	18 (72)	0.2
Age, years	39.6 ± 14.2	40.67 ± 13.07	37.7 ± 16.16	0.43
Retransplantation, *n* (%)	24 (34.8)	17 (38.6)	7 (28)	0.5
Time since dialysis, months	56.5 ± 65	60.1 ± 70.44	49.49 ± 54.4	0.51
Blood group, *n* (%) ^‡^				0.5
Type A	38 (55.1)	22 (50)	16 (64)	
Type B	6 (8.7)	4 (9)	2 (8)	
Type O	23 (33.3)	17 (38.6)	6 (24)	
Type AB	1 (1.4)	1 (2.2)	0 (0)	
**Donor**				
Age, years	20.9 ± 12.1	21.32 ± 12.25	20.04 ± 12.01	0.68
Deceased, *n* (%)	65 (94.2)	41 (93.1)	24 (96)	1
**Transplantation**				
Number of HLA A/B/DR mismatch	3.8 ± 1.4	3.59 ± 1.53	4.04 ± 1.17	0.18
Combined transplantation¶, *n* (%)	8 (11.6)	5 (11.4)	3 (12)	1
Cold ischemic time, minutes	934 ± 373	923 ± 401	954 ± 324	0.7
Delayed graft function, *n* (%)	12 (17.4)	8 (18.1)	4 (16)	1

*AMR, Antibody-Mediated Rejection; DSA, Donor-Specific antibodies. Unless noted otherwise results are expressed as mean ± standard deviation*.

* *Comparison between patients with sialylated DSA and patients with non-sialylated DSA (χ2 tests for comparison of proportions and unpaired t-test for comparison of continuous variables)*.

‡ *Data missing for 1 patient*.

*¶ kidney and pancreas*.

**Table 2:** Patient characteristics at the time of AMR diagnosis.

**Variable**	**Patients with AMR (*n* = 69)**	**Patients with di-sialylated DSA** **(*n* = 44)**	**Patients with un/mono sialylated DSA** **(*n* = 25)**	*p*-Value^*^
**CHARACTERISTICS OF AMR**				
**Clinico-biological characteristics**				
Time post-transplantation (months)	46.9 ± 51.6	47.4 ± 53	45.9 ± 50.2	0.2
Proteinuria (gram/day)	1.6 ± 4.5	1.07 ± 2.2	2.64 ± 6.91	0.28
Creatininemia (μmol/l)	295 ± 312.8	309.82 ± 273.82	268.96 ± 376.46	0.64
estimated GFR§ (ml/min/1.73 m^2^)	33.6 ± 20.4	28.8 ± 16.43	42.04 ± 24.08	**0.02**
Biopsy for protocol	9 (13)	3 (6.8)	6 (24)	0.1
Histological characteristics^**^				
Microvascular inflammation^†^	3.5 ± 1.2	3.55 ± 1.17	3.36 ± 1.19	0.53
Transplant glomerulopathy	1.6 ± 0.9	1.61 ± 0.92	1.52 ± 1	0.7
Interstitial Inflammation and Tubulitis	2.6 ± 2	2.55 ± 1.85	2.72 ± 2.37	0.75
Interstitial fibrosis and tubular atrophy	1.6 ± 0.8	1.68 ± 0.74	1.52 ± 0.87	0.44
Arteriosclerosis	1 ± 1.1	1.1 ± 1.17	0.84 ± 0.94	0.33
Endarteritis (vasculitis)	0.3 ± 5	0.2 ± 0.46	0.36 ± 0.57	0.23
**Immunological characteristics**				
IgG sialylation rate (%)§	1.8 (2.6)	1.87 (2.7)	1.77 (2.6)	0.15
DSA sialylation rate (%)	36 ± 37	57 ± 32	1 ± 2	**<0.001**
Types of DSA ^‡^				0.6
Preformed DSA, *n* (%)	12 (17.4)	9 (20.4)	3 (12)	
*De novo* DSA, *n* (%)	43 (62.3)	26 (59.1)	17 (68)	
Preformed + *de novo* DSA, *n* (%)	9 (13)	5 (11.4)	4 (16)	
**Classes of DSA**				
Class I, *n* (%)	22 (31.9)	14 (31.8)	8 (32)	0.7
Class II, *n* (%)	61 (88.4)	39 (88.6)	22 (88)	1
Class I + II, *n* (%)	14 (20.3)	9 (20.5)	5 (20)	1
Number of DSA	1.8 ± 1.2	1.8 ± 1.2	1.8 ± 1.1	1
MFI of immuno-dominant DSA	7606.1 ± 5767.5	8599.32 ± 5499.7	5858 ± 5093.16	**0.05**
C3d binding DSA, *n* (%)	40 (58)	27 (61.4)	13 (52)	0.6
**Treatments**				
Steroids pulses	60 (87)	41 (93.2)	19 (76)	0.1
Intravenous Immunoglobulins	1 (1.4)	1 (2.3)	0 (0)	1
Rituximab	38 (55.1)	27 (61.3)	11 (44)	0.3
Plasmapheresis	33 (47.8)	22 (50)	11 (44)	0.8

*AMR, Antibody-Mediated Rejection; DSA, Donor-Specific antibodies; MFI, Mean Fluorescence Intensity; GFR, Glomerular Filtration Rate. Unless noted otherwise results are expressed as mean ± standard deviation*.

* *Comparison between patients with sialylated DSA and patients with non-sialylated DSA (χ2 tests for comparison of proportions and unpaired t-test for comparison of continuous variables). § Calculated with the Modification of Diet in Renal Disease formula*.

** Banff scores (0: no significant lesion, 1: mild, 2: moderate, 3: severe)

† *Sum of the Banff scores for glomerulitis and capillaritis. § Approximation by OD(SNA)/OD(IgG) ratio*.

‡ *Data missing for five patients. Significant values (≤0.05) are marked in bold*.

**Figure 2:**
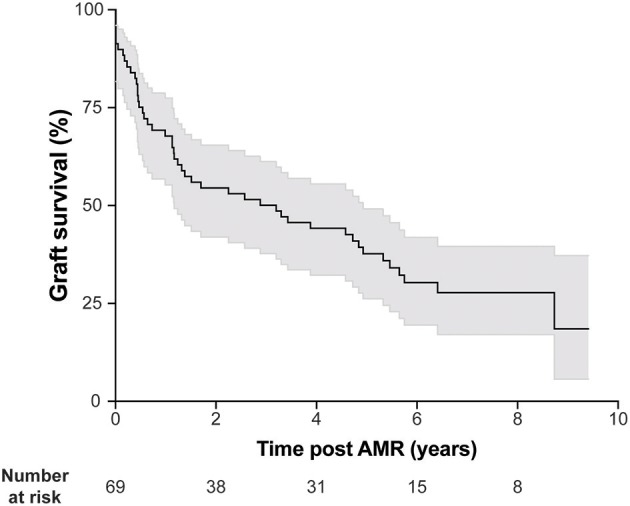
AMR is associated with heterogeneous kidney graft survival. Kidney graft survival of patients diagnosed with AMR. Gray shading indicates 95% confidence interval.

AMR was diagnosed 46.9 ± 51.6 months in average after transplantation. Consistent with the literature, kidney allograft survival after AMR diagnosis was highly heterogeneous (Figure 2), highlighting the fact that not all DSA have the same pathogenic potential.

### Purification of Sialylated IgG

**Figure 3:**
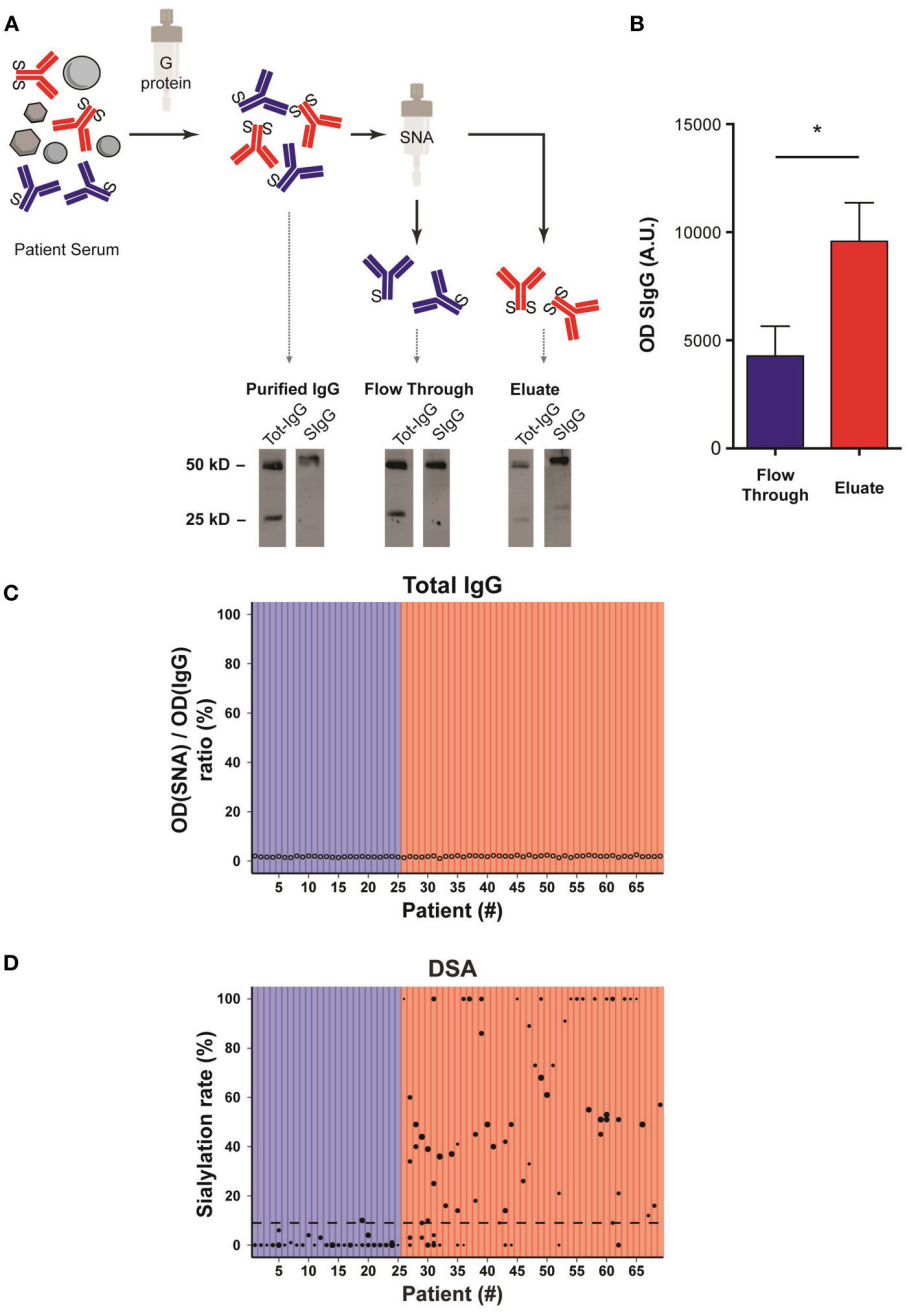
Sialylation status of the Fc fragment of DSA is variable. **(A)** Schematic representation of the method used to determine the sialylation status of IgG Fc fragments. Sera banked at the time of the diagnosis of AMR were passed through a G protein column and purified IgG were then passed on *Sambucus nigra lectin* (SNA) column that binds to sialic acid. The un/monosialylated IgG (blue) containing flow through and eluate (enriched in the di-sialylated IgGs, red), were collected separately and analyzed by western blot. **(B)** Western blot of the flow through (un/monosialylated IgGs) and the eluate (di-sialylated IgGs) were revealed with SNA, and the intensity of the 50 kD band, which corresponds to the IgG heavy chain (Fc fragment), was measured. ^*^p < 0.05; *t*-test. **(C,D)** Approximation of the total IgGs di-sialylated proportion (**C**, open circles,) and measurement of the di-sialylated DSA proportion (**D**, black circles), determined at the time of AMR diagnosis, are plotted for each patient (*n* = 69). **(D)** Each symbol is a distinct DSA specificity, the MFI of which is proportional to the size of the circle. The 69 transplanted patients are distributed into two groups depending on whether the pondered proportion of di-sialylated DSA was below (low DSA sialylation, *n* = 25; blue) or above (high DSA sialylation, *n* = 44; red) the median of di-sialylated DSA (9%, dashed line).

Purification of sialylated IgG was performed following the reference technique described by Kaneko et al. (20). Briefly, sera banked at the time of the diagnosis of AMR were passed through a G protein column and purified IgG were then passed on *Sambucus nigra lectin* (SNA) column that binds to sialic acid attached to terminal galactose in α-2,6 (Figure 3A).

Western blot analyses of the various serum fractions were conducted in reducing conditions and revealed with polyclonal anti-human IgG antibodies (Tot-IgG) and SNA (SIgG) to quantify, respectively, total and sialylated IgG. Sialic acid residues were mainly detected on 50 kD heavy chains, confirming that sialylation modifies the Fc region of IgG (Figure 3A).

Importantly, while the fraction eluted from SNA column showed a significant enrichment in sialylated IgG as compared with the flow through (flow through vs. eluate: 4270 ± 798 vs. 9581 ± 1262, *p* = 0.03; Figure 3B), the lectin blot of the latter fraction remained positive (Figure 3A). Our results therefore confirm the conclusion of Stadlmann et al. (23) that SNA column retain Fc regions with two sialic acid residues. In the rest of the MS we will therefore refer to un/mono-sialylated and di-sialylated IgG.

### The Proportion of Di-Sialylated DSA Is Highly Heterogeneous Across AMR Patients

The proportion of di-sialylated IgG, measured in the serum banked at the time of the diagnosis of AMR, showed remarkable homogeneity (median: 1.82%; range: 1.03–2.50%) across all the patients of the cohort (Figure 3C). This result is comparable to what previously reported in the literature for healthy volunteers (23).

The flow through (un/mono-sialylated IgG) and eluted (di-sialylated IgG) fractions from the SNA column of each AMR patients were screened in solid phase assay to quantify their respective content in anti-HLA antibodies. In contrast with total IgG, the proportion of di-sialylated DSA was highly variable from one patient to another and ranged from 0 to 100% (Figure 3D).

Based on these results, it was possible to distribute the 69 transplanted patients into two categories depending on whether the pondered proportion of di-sialylated DSA was < [group low DSA sialylation, “LowS-DSA”: *n* = 25 (36%)] or ≥ [group high DSA sialylation, HighS-DSA: *n* = 44 (64%)] to 9%, which represents the median % of di-sialylated DSA (dashed line; Figure 3D).

### Impact of DSA Sialylation on Clinical Outcome of AMR

The characteristics of the two groups of AMR patients are summarized in the right columns of Tables 1, 2. Baseline characteristics of donors and recipients were similar between the two groups at time of transplantation. Estimated Glomerular Filtration Rate (eGFR) at time of rejection diagnosis was lower in HighS-DSA group (28.8 ± 16.43 vs. 42.04 ± 24.08 ml/min; *p* = 0.02). Regarding DSA characteristics, HighS-DSA group had a significant higher MFI of immuno-dominant DSA (8599 ± 5499 vs. 5858 ± 5093; *p* = 0.05) but the sum of DSA MFI was similar in the two groups 12290 ± 10729 (HighS-DSA) vs. 9501 ± 10741 (LowS-DSA); *p* = 0.30). Treatment of AMR consisted of steroid pulses, intravenous immunoglobulins, plasmapheresis, or rituximab and was analogous in both groups.

**Figure 4:**
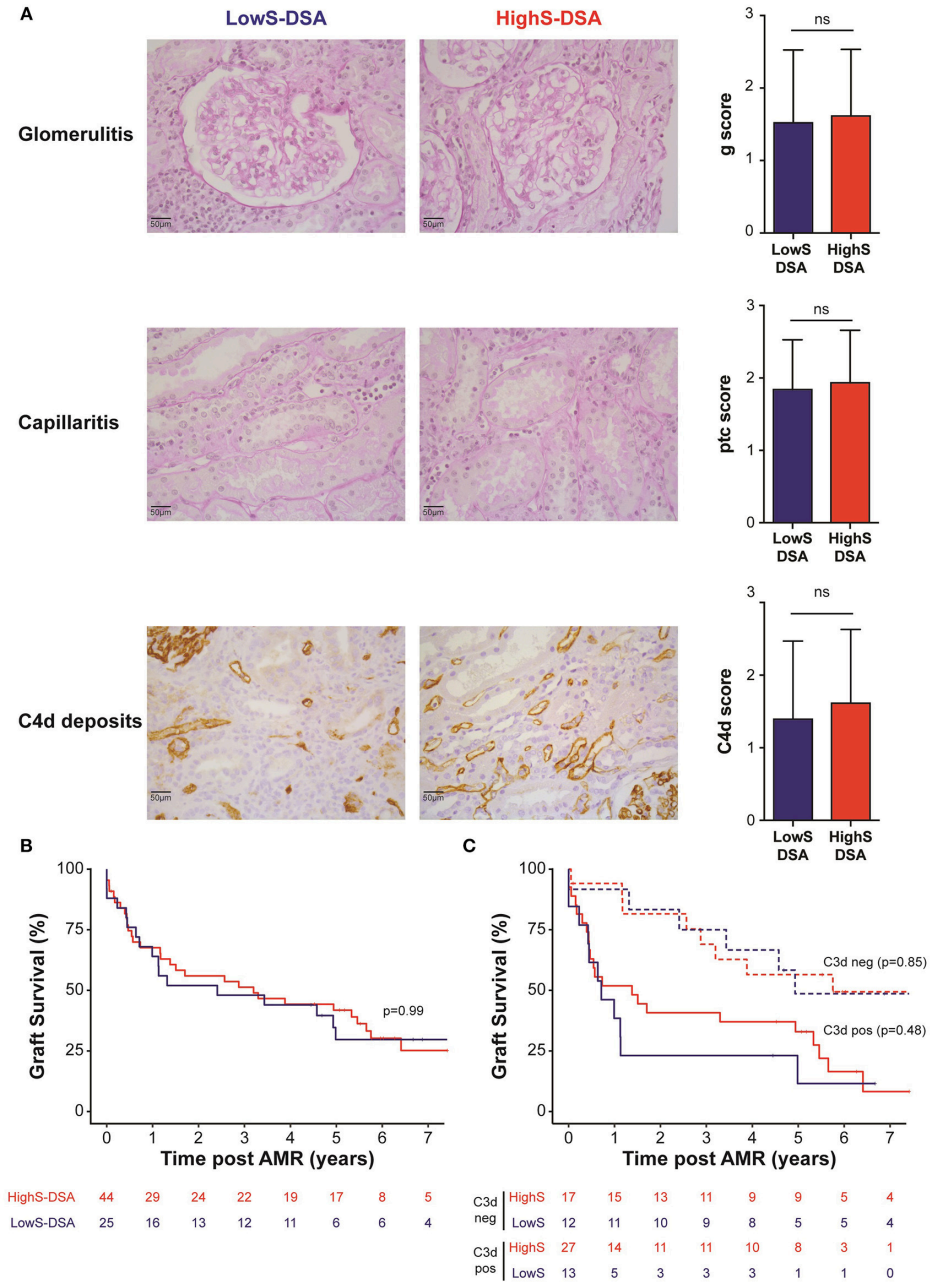
AMR outcome in LowS-DSA and HighS-DSA patients. **(A)** Histological findings of a representative patient from LowS-DSA (left column) and HighS-DSA (middle column) group are shown. Elementary lesions associated with AMR: glomerulitis (g, upper row), capillaritis (ptc, middle row), and complement deposition (C4d, lower row) were graded 0–3 according to Banff classification and compared between the two groups (right column). ns: *p* > 0.05; *t*-test. **(B,C)**. Kidney graft survival of LowS-DSA (blue) and HighS-DSA (red) patients were compared by log rank test for all AMR **(B)** or after stratification on C3d assay **(C)**.

Biopsies performed at time of AMR showed comparable histological lesions between LowS-DSA and HighS-DSA patients (Table 2), in particular regarding glomerulitis, capillaritis, and C4d Banff scores, known as the main features of AMR (Figure 4A). Allograft survival was not different in the two groups (Figure 4B), a result that remained unchanged when the AMR cases were split according to the ability of DSA to bind C3d (Figure 4C) or when different cut-off of di-sialylated DSA were examined (data not shown).

Because survival results could have been affected by the unequal distribution between LowS-DSA and HighS-DSA groups of eGFR at the time of rejection and MFI of immuno-dominant DSA (see above and Table 2), a Cox regression proportional hazard model was used to identify the variable independently associated with allograft survival. Exploratory univariate analysis identified 4 factors associated with allograft loss in the cohort (Table 3): eGFR at the time of rejection [hazard ratio [HR], 2.56; 95%; *p* = 0.002]; proteinuria at the time of rejection (HR, 2.51; *p* = 0.002); C3d antibody status (HR, 2.94; *p* = 0.001); and MFI of the immuno-dominant DSA (HR, 2.32; *p* = 0.005). Although the proportion of di-sialylated DSA was not associated with allograft loss in the univariate analysis (*p* = 0.27), the variable was integrated in the multivariate analysis along with the 4 previously identified variables. Only three factors were independently associated with allograft loss at diagnosis of AMR: (i) eGFR at the time of rejection (HR, 2.25; *p* = 0.008); (ii) proteinuria at the time of rejection (HR, 2.36; *p* = 0.004); and (iii) C3d binding status of immuno-dominant DSA (HR, 2.12; *p* = 0.03).

**Table 3:** Univariate and multivariate analyses of risk factors for death-censored allograft loss.

		**Univariate**			**Multivariate**		
**Variable**	**No. of patients**	**HR**	**95% CI**	***P*-Value**	**HR**	**95% CI**	***P*-Value**
**CLINICO-BIOLOGICAL FACTORS**							
**Recipient gender**							
Female	27	1.00	Reference				
Male	42	1.04	(0.58–1.90)	0.89			^†^
Recipient age (per 1 year increment)	69	0.99	(0.97–1.01)	0.18			^†^
**Retransplantation**							
No	45	1.00	Reference				
Yes	24	1.02	(0.56–1.84)	0.96			^†^
**Donor type**							
Living	4	1.00	Reference				
Deceased	65	0.78	(0.24–2.52)	0.78			^†^
Donor age (per 1 year increment)	69	1.00	(0.98–1.02)	0.90			^†^
**Number of mismatches A/B/DR**							
≤3	20	1.00	Reference				
>3	49	0.80	(0.44–1.48)	0.48			^†^
Cold-ischemia time per 1-min increment	69	1.00	(0.99–1.00)	0.23			^†^
**Estimated GFR at the time of rejection^§^ (ml/min/1.73 m**^**2**^**)**							
≥30	36	1.00	Reference				
<30	33	**2.56**	(1.43–4.57)	**0.002**	**2.25**	(1.23–4.10)	**0.008**
**Proteinuria at the time of rejection (grams/day)**							
<0.5	43	1.00	Reference				
≥0.5	26	**2.51**	(1.41–4.49)	**0.002**	**2.36**	(1.32–4.25)	**0.004**
HISTOLOGICAL FACTORS^*^							
Microvascular inflammation^**^							
2 or 3	34	1.00	Reference				
≥4	35	1.28	(0.72–2.27)	0.40			^†^
**C4d graft deposition**^‡^							
0 or 1	26	1.00	Reference				
≥2	41	1.15	(0.64–2.09)	0.64			^†^
**Interstitial inflammation and tubulitis**							
0 or 1	27	1.00	Reference				
≥2	42	1.28	(0.70–2.33)	0.43			^†^
**Transplant glomerulopathy**							
0 or 1	49	1.00	Reference				
≥2	20	0.95	(0.51–1.77)	0.87			^†^
**Endarteritis (vasculitis)**							
0	54	1.00	Reference				
≥1	15	1.17	(0.59–2.31)	0.65			^†^
**Arteriosclerosis**							
0 or 1	52	1.00					
≥2	17	0.77	(0.39–1.51)	0.44			^†^
**Interstitial fibrosis and tubular atrophy**							
0 or 1	29	1.00	Reference				
≥2	40	**1.55**	(0.85–2.83)	0.15			^†^
**IMMUNOLOLOGICAL FACTORS**							
**C3d-binding DSA**							
No	29	1.00	Reference				
Yes	40	**2.94**	(1.56–5.54)	**0.001**	**2.12**	(1.09–4.11)	**0.03**
**Sialylated DSA**							
No	38	1.00	Reference				
Yes	31	1.37	(0.78–2,41)	0.27	–	–	ns
**MFI immuno-dominant DSA**							
<6000	34	1.00	Reference				
≥6000	35	**2.32**	(1.30–4.18)	**0.005**	–	–	ns
**Number of DSA**							
1	38	1.00	Reference				
≥2	31	1.26	(0.71–2.22)	0.43			^†^

*HR, Hazard Ratio; CI, Confidence Interval; ns, Not Significant; GFR, Glomerular Filtration Rate; DSA, Donor-Specific antibodies; MFI, Mean Fluorescence Intensity; No, number*.

* *Banff scores (0: no significant lesion, 1: mild, 2: moderate, 3: severe)*.

** *Sum of the Banff scores for glomerulitis and capillaritis*.

‡ *Data missing for two patients. Variables at the P-level <0.1 in univariate model and the sialylated DSA variable were incorporated into the multivariate model*.

† *the variables that were not tested in multivariate model*.

§ *Calculated with the Modification of Diet in Renal Disease formula. Variables with significant p-values (≤0.05) are marked in bold*.

### Does Sialylation of Fc Fragment Really Matter for IgG Function?

To validate these clinical findings, which are, by essence, only correlative, we undertook an *in vitro* study aiming at directly assessing the impact of sialylation status of the Fc fragment of IgGs on their ability to (i) activate the classical complement cascade, and (ii) trigger activation of NK cells, the two mechanisms involved in graft destruction during AMR (12, 26).

**Figure 5:**
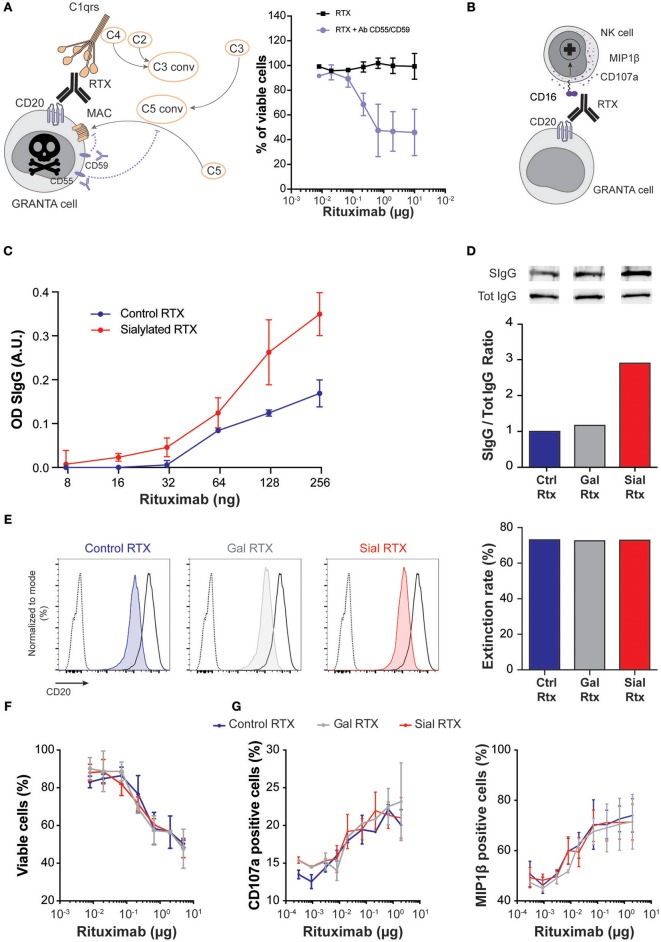
Sialylation of Fc fragment does not modify IgG function. **(A,B)** Schematic representation of the experimental models used to assess the ability of the human monoclonal IgG1 rituximab (RTX) to trigger complement-dependent cytotoxicity **(A)** and antibody-dependent activation of NK cells **(B)**. Complement-dependent cytotoxicity assay **(A)** with human serum required blocking the surface regulatory proteins CD55 and CD59, which prevent the terminal polymerization of the membrane attack complexes. Commercially available rituximab (control rituximab, Ctrl RTX; blue) was first galactosylated (Gal RTX; gray) before sialic acid residues were added on galactose residues (Sial RTX; red). The success of chemoenzymatic glycosylation of RTX was controlled by ELISA using SNA **(C)** and by western blotting **(D)**. **(E)** Competitive binding assay was used to compare the antigen-binding capacity of the different glycosylated forms of RTX. Overlay of the flow cytometry profiles of 10^5^ CD20-expressing GRANTA cells incubated with (i) PBS (dotted line), (ii) saturating amount of FITC conjugated Ctrl RTX (solid black line), and (iii) saturating amount of FITC conjugated Ctrl RTX after prior incubation with 2 μg of Ctrl RTX (blue, left panel), or 2 μg of Gal RTX (gray, middle panel), or 2 μg of Sial RTX (red, right panel). The reduction of MFI (extinction rate) observed when GRANTA cells were pre-incubated with glycosylated forms of RTX as compared with GRANTA cells incubated directly in AF488-conjugated Ctrl RTX was expressed as a percentage and plotted (right histogram). **(F)** The ability of the three glycosylated forms of RTX to trigger the death of CD20-expressing GRANTA cells was compared in human complement-dependent cytotoxicity assay. **(G)** The ability of the three glycosylated forms of RTX to trigger the degranulation (left panel) and the production of chemokine (right panel) by human NK cells was compared.

For each of these two pathophysiological mechanisms, an experimental model was designed (Figures 5A,B), in which the functionality of the same chimeric mouse-human IgG1 monoclonal antibody (rituximab, RTX) was compared according to the glycosylation status of its Fc fragment. The major Fc glycans of commercial RTX are core-fucosylated biantennary complex-type oligosaccharides lacking sialic acid (22). Commercially available RTX (which was used as control, Ctrl-RTX; blue) was first galactosylated (Gal-RTX; gray) before sialic acid residues were added on galactose residues (Sial-RTX; red). The success of sialylation procedure was demonstrated by the increased binding of SNA to Sial-RTX coated wells observed in ELISA (Figure 5C). Western blotting with polyclonal anti-human IgG antibodies (Tot-IgG) and SNA (SIgG) confirmed that sialylated form of RTX was enriched by 3-folds in Sial-RTX as compared with Ctrl-RTX and Gal-RTX (Figure 5D). Chemoenzymatic glycosylation remodeling of the Fc fragment of RTX did not impact on its ability to bind to its antigenic target (human CD20) as demonstrated in competitive binding assay (Figure 5E).

No difference was observed regarding the ability of Ctrl-RTX, Gal-RTX, and Sial-RTX to trigger the death of CD20-positive GRANTA cells in human complement-dependent cytotoxicity assay (Figure 5F). The three glycosylated forms of RTX were also equally effective to activate degranulation of human NK cells and their production of chemokine (Figure 5G). We concluded that sialylation status of Fc fragment neither impacts IgG's ability to trigger classical complement activation, nor to bind to Fcγ receptors of innate immune effectors.

## Discussion

In the present translational study, we analyzed a cohort of deeply phenotyped renal transplant recipients diagnosed with AMR and observed that the sialylation status of donor-specific antibodies (DSA) was highly variable.

The heterogeneity in sialylation status of the Fc fragment of DSA was evident not only between patients but also for the different DSA specificities of the same individual. This variable was neither affected by the fact that the alloantibody was preformed (vs. *de novo*), the nature of the HLA target (class I vs. II molecules), nor the diversity of alloantibody repertoire. In fact, extensive analysis only identified eGFR as clinical variable associated with lower level of DSA sialylation. Although this finding is consistent with old observations that chronic renal failure correlates with increased activity of sialic acid transferase (27, 28), the latter explanation does not clarify why the same difference was not observed for the rest of circulating IgGs (the sialylation level of which was remarkably similar across the patients). Furthermore, the lack of clear relation between eGFR and the proportion of sialylated DSA in a linear regression model (y = −0.0048x + 0.526; *r*^2^ = 0.069) is also a clue that this difference might just have been observed by random luck.

Because sialylation alters the Fc portion of antibody, which plays a crucial role in recruiting the complement cascade component C1q and/or the binding to the Fcγ receptors of innate immune effectors, we have put forward the hypothesis that this overlooked characteristic of DSA may partly explain the heterogeneity of clinical outcome in AMR (9, 15, 29). The concept that (beyond the quantity/titer of alloantibodies) the qualitative characteristics of the Fc fragment also impact DSA pathogenic potential was supported by recent publications demonstrating that patients whose DSA were IgG2 or IgG4 (two heavy chain isotypes with weak complement-binding ability) had better allograft survival (30, 31). Furthermore, decreased levels of total IgG sialylation have been shown to correlate with more active forms of various auto-immune diseases, including systemic lupus erythematosus (32, 33), inflammatory bowel disease (34) and granulomatosis with polyangiitis (35). Conversely, increase in IgG Fc sialylation has been associated with clinical remission in chronic inflammatory demyelinating polyneuropathy (36) and rheumatoid arthritis (37).

Our study however, did not find any correlation between AMR severity and DSA sialylation status. Indeed, both the intensity of histological lesions and graft survival was similar in LowS-DSA and HighS-DSA groups. Moreover, the proportion of di-sialylated DSA was not associated with graft loss in multivariate analysis. Despite the relatively limited number of patients enrolled in this study, it seems unlikely that this result was merely explained by a lack of statistical power because the multivariate analysis did identify the variables usually associated with detrimental outcome in AMR: i.e., eGFR and proteinuria at the time of rejection, and C3d binding status of immuno-dominant DSA.

It should be mentioned that whether (and how) sialylation of the Fc fragment of an antibody impacts on its functions remains a highly controversial debate. Independent groups have reached opposite conclusions regarding the relation between Fc sialylation and the ability of IgG to trigger ADCC [(38) vs. (21)] and complement activation [(22) vs. (39)]. Therefore, in order to validate our clinical observations, we moved to experimental approach. Although, *in vitro* models may be viewed as over-simplistic to reproduce the complex pathophysiology of AMR, they permitted to directly assess the impact of the Fc fragment sialylation status on the function of a particular monoclonal IgG. In line with our clinical results, the enzymatic addition of sialic acid residues on the Fc fragment of rituximab neither increased its ability to activate the classical complement cascade nor to trigger the activation of NK cells, the two crucial mechanisms involved in graft destruction during AMR (12, 26).

We conclude that, although the Fc fragment sialylation status of DSA is highly variable, this characteristic does not seem to have a significant impact on DSA pathogenicity and cannot be used to stratify the risk of graft loss in AMR.

## Data Availability

The datasets generated for this study are available on request to the corresponding author.

## Author Contributions

OT and SB designed the study. OT, EM, SB, AK, AS, and EP took care of patients and provided clinical data. AK, EP, and AS collected the data and built the database. OT and TB analyzed the data. JH, SD, VM, ED, SB, DC, and VD performed the *in vitro* analyses. SD, TB, MR, and OT made the figures and tables. JH, TB, SD, TD, VD, and OT drafted and/or revised the paper. All authors approved the final version of the manuscript.

### Conflict of Interest Statement

The authors declare that the research was conducted in the absence of any commercial or financial relationships that could be construed as a potential conflict of interest.
